# Current status of radiologist staffing, education and training in the 27 EU Member States

**DOI:** 10.1186/s13244-025-01925-7

**Published:** 2025-03-15

**Authors:** Adrian P. Brady, Graciano Paulo, Boris Brkljacic, Christian Loewe, Martina Szucsich, Monika Hierath, on behalf of the European Society of Radiology

**Affiliations:** 1 Department of Radiology, University College Cork, Cork, T12 AK54 Ireland; 2 Polytechnic University of Coimbra. IPC-ESTESC—Coimbra Health School, Medical Imaging and Radiotherapy, Coimbra, Portugal; 3 H&TRC—Centro de Investigação em Saúde e Tecnologia, Lisbon, Portugal; 4 Department of Diagnostic and Interventional Radiology, University Hospital Dubrava, University of Zagreb School of Medicine, Zagreb, Croatia; 5 Division of Cardiovascular and Interventional Radiology, Department of Biomedical Imaging and Image-Guided Therapy, Medical University of Vienna, Vienna, Austria; 6 European Society of Radiology, Vienna, Austria

**Keywords:** Ionising radiation, Radiation protection, Workforce, Education and training

## Abstract

**Abstract:**

This second article of a series of three publications summarises the radiologist situation regarding staffing as well as education and training as analysed by The European Union Radiation, Education, Staffing & Training (EU-REST) study. Despite certain limitations posed by the dependence on survey responses, the results demonstrate that, for both workforce and education/training, considerable heterogeneity exists between Member States, which will impact healthcare delivery and the level of knowledge, skills, and competencies available. The number of radiologists per million inhabitants varies from 51 to 270. 16 out of 27 Member States have Radiologist numbers below the EU average of 127, and 45% of Radiologists in Europe are over 51 years old (in 2022). Clear guidance and metrics about workforce availability for the professions involved in the use of ionising radiation are needed to secure and improve the quality of healthcare delivery in Europe. Although the main scope of the EU-REST study was education, training and workforce availability, an attempt was made to characterise the numbers of pieces of medical imaging and radiotherapy equipment.

**Critical relevance statement:**

Clear guidance and metrics on radiologist staffing and education/training are needed to address workforce shortages and harmonise education and training standards across the EU-27.

**Key Points:**

- The article describes the radiologist situation regarding staffing and radiation protection education in the EU Member States.
- Radiologist staffing and training vary considerably across the EU-27.
- The fact that more than half of the EU Member States have radiologist numbers below the EU average, and the large proportion of radiologists over 51 years of age, show that clear guidance and metrics are needed to ensure future quality of radiological care.

In Part 1 [1], we summarised the genesis, structure, and conduct of the EU-REST project [2], commissioned by the European Commission to identify the current status of workforce availability, education and training in the 27 European Union Member States (EU 27) in the professional groups involved in medical applications of ionising radiation. The project was funded by the EU4Health Programme of the EU [3]; it was intended to form part of the actions of the Strategic Agenda for Medical Ionising Radiation Applications (SAMIRA) Action Plan, and to contribute to the implementation of Europe’s Beating Cancer Plan [4].

In this second (of three) article, we will outline the findings of the earlier stages of the project with respect to the current status of these parameters, as they apply to radiologists.

The work reported here constituted the earlier work packages of the project. We surveyed all identified relevant stakeholders [1] in the EU 27, encompassing appropriate authorities and professional bodies, national professional societies, radiation protection authorities and medical associations/chambers, with respect to the current status of workforce availability, and education and training of the relevant professional groups within each country.

We also identified (by means of an extensive literature search) and analysed any available guidelines already in existence overing the subject areas of the project.

Finally, we analysed the responses to the Main Survey to define the current status of education, training and workforce availability. This analysis will form the bulk of this article.

In Part 3 [5], we will report on the guidelines for education, training and workforce availability for radiologists which were developed as a result of the EU-REST project, building on the information collected about present circumstances, as reported in this article.

## Data collection and analysis

The Pre-Survey (described in Part 1 [1]) was designed to ensure that the Main Survey was circulated to any and all potential sources of useful information regarding education, training and workforce availability. The Pre-Survey asked respondents to identify the bodies and/or agencies in their countries that had responsibility for determining and implementing curricula, education and training standards and duration, and workforce determination and maintenance, with a view to using the contacts identified as the appropriate recipients for the Main Survey. A total of 109 responses were received to the Pre-Survey, with at least one from each of the 27 EU Members, generating a list of 273 contacts of relevant authorities and bodies in the EU 27 responsible for staffing, education and training issues.

The Main Survey consisted of 458 questions in total, including answer options from drop-down menus and free-text options, as appropriate. Questions were grouped to obtain information indicating the origins of responses (respondent’s demographics, the groups/organisations/professions on whose behalf responses were submitted, etc.), followed by detailed questions about education and training (both primary and continuing) for their professional group. A separate section of the survey collected information on workforce availability, demographics, recruitment and planning for the professional group for which responses were being provided. Where relevant, information about equipment availability was sought. The final sections of the survey asked about quality and safety standards and structures and invited respondents to provide information about any guidelines used within their countries relating to the matters under review. Respondents were not required to answer every question; depending on the profession for which answers were being given, and depending on the specific competence of respondents, pathways existed to circumvent many of the questions. We estimated that the survey would take 20 to 30 minutes for each respondent to complete.

PDFs of the Pre-Survey and Main Survey are included in Supplementary Material.

Support for data analysis was provided by a statistician, to ensure accuracy and consistency of conclusions.

The Main Survey was implemented, in English, in Survey Monkey. It was tested by 24 consortium member representatives for functionality, and, after optimisation, was distributed in December 2022 to the stakeholders and recipients identified through the Pre-Survey and the stakeholder mapping exercise described in Part 1 [1]. The survey was sent to approx. 270 contacts of national organisations, competent authorities as well as EU27 national professional societies for Radiology (ESR), Nuclear Medicine (EANM), Radiotherapy (ESTRO), Radiography (EFRS) and Medical Physics (EFOMP), who were asked to distribute the survey to their members. Likewise, HERCA was asked to send the survey to the EU27 national radiation protection authorities, and the UEMS to send it to the EU27 national medical associations/chambers.

A total of 186 survey responses were received from all 27 EU Member States, the majority coming from national professional and scientific societies, with a minority from national authorities. The proportion of Member States submitting responses varied among the professional groups. With respect to medical specialties, the highest number of Member State responses received was from radiologists, with submissions from 23 of the EU 27 (an 85% response rate for this group). A total of 38 responses was received regarding radiologists, with multiple responses from a minority of countries.

A data cleaning process was undertaken by consortium members following receipt of responses. This was not intended to verify accuracy or correctness of responses, but was designed to maximise completeness and coherence of responses where multiple overlapping responses to the survey had been received from any given country and/or professional group. Additionally, personal contacts with respondents were made by the consortium members responsible for data analysis in some cases, to obtain missing data and to clarify any ambiguities. At the end of the data cleaning process, the usable responses regarding radiologists had decreased by 1, with data analysis performed on data from 22 (81%) Member States.

Supplementary Tables S1 and S2 list the response rates (and sources) for each professional group from each of the 27 EU Member States.

**Fig. 1:**
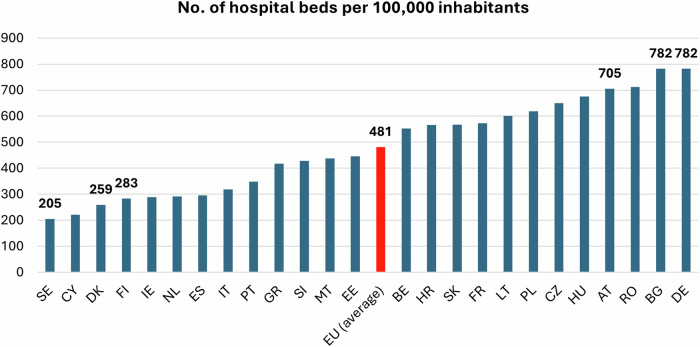
No. of hospital beds per 100,000 inhabitants here. Country codes: AT—Austria, BE—Belgium, BG—Bulgaria, HR—Croatia, CY—Cyprus, CZ—Czechia, DK—Denmark, EE—Estonia, FI—Finland, FR—France, DE—Germany, GR—Greece, HU—Hungary, IE—Ireland, IT—Italy, LT—Latvia, LV—Lithuania, MT—Malta, NL—Netherlands, PL—Poland, PT—Portugal, RO—Romania, SK—Slovakia, SI—Slovenia, ES—Spain, SE—Sweden

Analysis was also assisted by the collection of data on Member State population, numbers of hospitals, and numbers of hospital beds per 100,000 inhabitants (Fig. 1), derived from OECD.STAT data.

Thirteen EU countries have a lower number of hospital beds than the EU average (481), with Sweden, Cyprus and Denmark amongst those with the lowest numbers (< 260/100,000). Bulgaria and Germany have the highest number of hospital beds (> 750/100,000).

## Radiologists in Europe

### Workforce

According to the results from the Main Survey, there are 60,771 radiologists in Europe, with a ratio of 127 radiologists per 1,000,000 inhabitants. For the countries that provided the age profile (*n* = 17), approximately 19% (8356) of radiologists will retire in the next 5 years and 45% are over 51 years old (these results reflect the position in 2022).

Those countries whose workforce is older will lose a higher proportion of their radiologists to retirement in a relatively short number of years. Replacement due to this attrition should be planned for when determining trainee numbers.

**Fig. 2:**
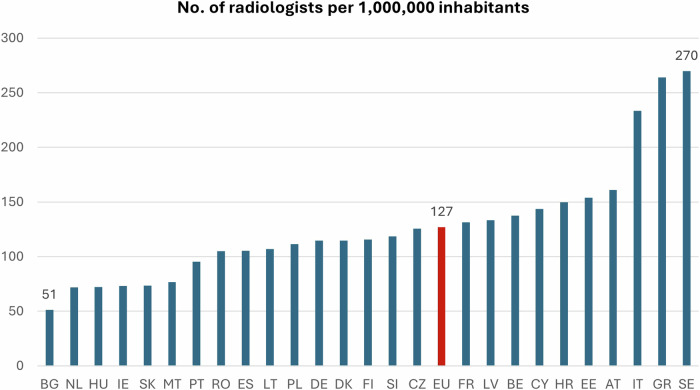
Number of radiologists per 1 million inhabitants

The number of radiologists per million inhabitants varies significantly between Member States, Bulgaria having the lowest number (51/M) and Sweden the highest (270/M). The EU average number is 127/M (Fig. 2).

In Table 1, standardised population results (per 1,000,000 inhabitants) are shown to facilitate the comparison between countries with EU average, median, min, max, and *dif factor (ratio between max and min values)*.

**Table 1:** Standardised workforce overview per 1 million inhabitants

Country	No. of radiologists per 1 million inhabitants
AT	161
BE	138
BG	51
HR	150
CY	144
CZ	126
DK	115
EE	154
FI	116
FR	131
DE	115
GR	264
HU	72
IE	73
IT	233
LT	133
LV	107
MT	77
NL	72
PL	112
PT	95
RO	105
SK	74
SI	119
ES	105
SE	270
EU mean	127
# countries lower than mean	16
EU median	115
# countries lower than median	13
min	51.2
max	269.8
*dif factor*	5.3

The highlighted cells in each country line indicate that the value is lower than the EU average (16 countries for Radiologists).

**Fig. 3:**
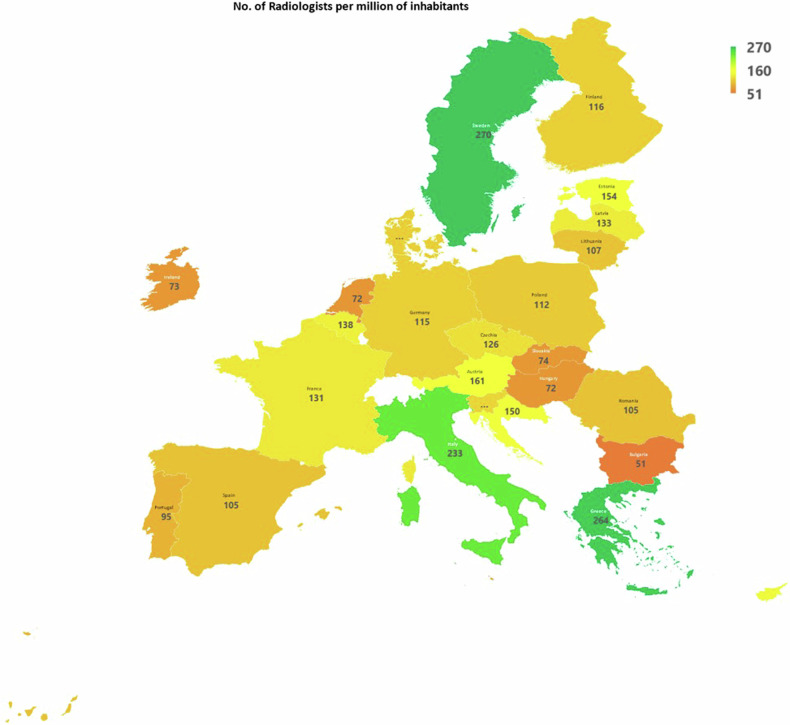
Geographical distribution of radiologists

The colour map in Fig. 3 shows the geographical distribution of radiologists across Europe, identifying the 16 countries with a density of radiologists lower than the EU average (dark orange) and the 10 above the EU average, with IT, GR and SE (green) having a significantly higher number than other countries.

As an exercise to assist comparison of workforce distribution across different countries, 5 EU countries which have approximately the same population (10 M) are compared and their workforce distribution is analysed: CZ, GR, HU, PT, SE (Table 2).

**Table 2:** Number of radiologists in 5 countries with similar population

Country	No. of radiologists
CZ	1321
GR	2800
HU	700
PT	987
SE	2820

This table clearly shows the huge heterogeneity in the numbers of radiologists available in these 5 countries, the difference between the lowest and the highest value amounting to a factor of ≈ 4.

With respect to the age profile of the available radiology workforce, there are nine countries (HR, CZ, EE, FR, HU, IT, LT, PL, SE) which will lose a higher share of the workforce to retirement in the 5 years from 2022 than the EU average (19%), assuming a retirement age of 66 years. LT presents the highest value (35%)—see Table 3.

**Table 3:** Radiologists’ age profile

Country	Retirement in 5 years	%	< 50 years old	> 51 years old
AT	145	10%	60%	40%
BE				
BG	18	5%	75%	25%
HR	128	22%	56%	44%
CY				
CZ	343	26%	43%	57%
DK				
EE	49	24%	41%	59%
FI				
FR	1960	22%	39%	61%
DE				
GR	280	10%	55%	45%
HU	140	20%	45%	55%
IE	37	10%	60%	40%
IT	2800	20%	50%	50%
LV				
LT	105	35%	45%	55%
MT	2	5%	90%	10%
NL	189	15%	60%	40%
PL	882	21%	51%	49%
PT				
RO				
SK				
SI	45	18%	57%	43%
ES	500	10%	65%	35%
SE	733	26%	47%	53%
EU	8356	19%	55%	45%

It is important to highlight the fact that in CZ, EE, FR, HU, IT, LT, and SE more than 50% of radiologists are over 51 years old. Among these countries, special attention should be given to countries such CZ, HU, LT, which have overall radiologist numbers per million inhabitants lower than the EU average.

When questioned if there are sufficient qualified practitioners to fill all available vacancies, 3 countries (DE, IE, ES) replied “no”.

### Education/training

Specialty training in radiology varies from 4 to 6 years, with an average of 4.9 years. Specific training in radiation protection during overall radiology training varies from 2 weeks or less to up to 16–24 weeks; the majority of countries (13) require specific certification in Radiation Protection, with mandatory continuous professional development in 8 countries (Table 4).

**Table 4:** Training requirements for radiologists

Country	Speciality training (years)	Training in RP (weeks)	Specific certification required in RP?	CPD in RP mandatory
AT	5.25	2 or less	Yes	Yes
BE	5.00		Yes	Yes
BG	4.00	2 or less	Yes	
HR	5.00	2 or less	No	Yes
CY	na	na	na	na
CZ	5.00	na	No	na
DK	5.00	na	No	na
EE	4.00	4–12	No	Yes
FI	5.00	2–4	Yes	na
FR	5.00	na	Yes	Yes
DE	5.00	Don’t know	Yes	Yes
GR	4.50	2–4	No	na
HU	5.00	2 or less	Yes	Yes
IE	5.00	2–4	No	na
IT	4.00	4–12	No	na
LV	na	na	na	na
LT	6.00	none	Yes	na
MT	5.00	2–4	No	na
NL	5.00	2–4	Yes	No
PL	5.00	2 or less	Yes	na
PT	na	na	na	na
RO	5.00	16–24	Yes	na
SK	5.00	2 or less	Yes	na
SI	5.00	2–4	Yes	Yes
ES	4.00	2–4	No	na
SE	5.50	na	No	na

*na* no answer

## Medical imaging equipment availability in Europe

Although it was not a primary focus of the EU-REST project, we felt that it would be helpful to also collate data, where possible, about the availability of medical imaging equipment in EU Member States. Available official data about equipment availability (EUROSTAT and OECD reports—insert refs) are inconsistent, potentially leading to erroneous analysis and conclusions if relied upon.

Through the Main Survey of this project, an attempt was made to collate equipment availability information, but unfortunately, the data received were limited, and, to some extent, also inconsistent with the official reports available, showing that there is an urgent need for the European Commission to support the development of a strategy to implement a central registry, to allow realistic and real-time access to this very important information, in order to assist health policymakers to make decisions based on reliable data. Nevertheless, the study consortium considered it relevant to present the data obtained through the survey (when available) and to complete it with the data from EUROSTAT.

Data for diagnostic radiology equipment includes CT, MRI, Plain radiography, Mammography, Mobile Radiology, and Angiography/Interventional suites. Table 5 gives an overview of available equipment (from data reported in the Main Survey, and otherwise from EUROSTAT. The asterisks (“*”) in the table represent countries with data extracted from EUROSTAT), including the numbers of pieces of equipment per million inhabitants, to allow easier comparison among countries. CT, MRI and Mammography equipment are the modalities with the greatest amount of data available. The highlighted cells in the tables for these types of equipment correspond to the countries with numbers of pieces of equipment lower than the EU average.

**Table 5:** Diagnostic radiology equipment

Country	Population (EUROSTAT)	CT scanners	CT scanners/ mio. inhab.	MRI scanners	MRI scanners/ mio. inhab.	Diagnostic radiography units	Diagnostic radiography units/ mio. inhab.	Mammography units	Mammography units/ mio. inhab.	Mobile radiology units	Mobile radiology units/ mio. inhab.	Angiographic/interventional suites	Angiographic/interventional suites/ mio. inhab.
AT	8,978,929	233	26	178	20								
BE	11,631,136	283	24	134	12	1342	115	445	38	1009	87	221	19
BG	6,838,937	281	41	80	12			214	31				
HR	3,879,074	95	24	65	17	418	108	147	38	168	43	50	13
CY	1,244,000	34	27	18	14			52	42				
CZ	10,516, 707	193	18	118	11	1728	164	113	11	888	84	744	71
DK	5,873,420	173	29	85	14	456	78	96	16	307	52	68	12
EE	1,331,796	28	21	22	17			15	11			11	8
FI	5,548,241	94	17	169	30			171	31				
FR	67,842,582	1285	19	1130	17								
DE	83,237,124	1586	19	1072	13			405	5				
GR	10,603,810	468	44	359	34			736	69				
HU	9,689,010	203	21	144	15	627	65	198	20			27	3
IE	5,060,005	101	20	78	15			82	16				
IT	59,983,122	2229	37	1857	31			2098	35				
LT	1,884,000	71	38	30	16			53	28				
LV	2,805,998	87	31	40	14			51	18				
MT	520,971	9	17	5	10			6	12			2	4
NL	17,590,672	256	15	233	13								
PL	37,654,247	928	25	441	12	3908	104	595	16			1950	52
PT	10,361,831	189	18	107	10			129	12				
RO	19,038,098	368	19	227	12			171	9				
SK	5,434,712	114	21	62	11	2101	387	89	16			48	9
SI	2,107,180	40	19	28	13			31	15				
ES	47,432,805	949	20	863	18			765	16				
SE	10,452,326	293	28	191	18			198	19				
	447,540,733	10,590	25	7736	16	10,580	146	6860	23	2372	67	3121	21

Data extracted from the survey and from EUROSTAT reveal that there are 10,590 CT scanners in Europe, with an average of 25 CTs per million inhabitants.

The great majority of the surveyed countries (17) show values below that number. Greece (44) and Bulgaria (41) are the countries with the highest numbers of CT scanners. Netherlands (15) has the lowest number of CT scanners per million inhabitants.

There are 7736 MRI scanners in Europe, with an average of 16 MRIs per million inhabitants. The great majority of countries (17) show values below that number. Greece (34), Italy (31) and Finland (30) are the countries with the highest numbers of MRI scanners. Portugal (10) has the lowest number of MRI scanners per million inhabitants.

There are 6860 mammography units available in Europe, with an average of 23 units per million inhabitants. The majority of countries (14) show values below that number. Greece (69) is the country with the highest number of units available by far. Germany (5) has the lowest number of mammography units per million inhabitants.

Considering the limited data for the other modalities, it is not possible to make specific analyses.

## Discussion

To our knowledge, this is the first study aimed at characterising:

- the workforce availability of health professionals involved in the use of ionising radiation for diagnostic and therapeutic procedures and
- the corresponding education and training in radiation protection.

This article specifically reports on these data as they apply to Radiologists. Other articles will report on the EU-REST project data and outcomes as they apply to other professional groups included in the project (Nuclear Medicine Physicians, Radiation Oncologists, Radiographers, Medical Physicists/ Medical Physics Experts, and Radiation Therapists).

Our results clearly demonstrate that for both workforce and education/training, there is huge heterogeneity between Member States and professions, which will obviously have an impact on healthcare delivery and the level of knowledge, skills and competences available, in terms of both radiation protection and of professional service delivery in general.

For Radiologists, the number of professionals per million inhabitants varies from 51 (Bulgaria) to 270 (Sweden), with the EU average being 127. There is a lack of evidence to explain the reasons behind this huge heterogeneity. Some of the causes may be associated with the type of organisation of each country’s healthcare system and practice (private, public or mix of both), the existence of teleradiology practice and the fact that in some countries, there is a role extension, with radiologists also being responsible for activities in nuclear medicine.

Regardless of the underlying reasons for the heterogeneity, the fact that 16 out of 27 Member States have Radiologist numbers below the EU average is disturbing, suggesting a failure to plan adequately for future needs, particularly considering continuing year-on-year increasing utilisation of radiology services.

The fact that 45% of Radiologists in Europe are over 51 years old (in 2022) emphasises the urgent need to set in place an action plan to attract younger generations into this medical specialty.

The results show that clear guidance and metrics about workforce availability for the professions involved in the use of ionising radiation is needed, as a tool to harmonise the access of patients to these professionals in Europe, thereby contributing to overall improvement of the quality of healthcare delivered. The lack of such guidance and metrics, and also of standards of practice, makes it difficult to define good practices from existing models.

Radiologist speciality training in Europe is (to some extent) harmonised; however, education and training (E&T) in radiation protection (RP) shows large variations (from less than 2 weeks to 24 weeks). In most countries, specific certification in radiation protection is required, but few answers were provided to the question asking “if CPD in RP is mandatory”, making statistical analysis of this particular parameter invalid.

Although the European Directive 2013/51/EURATOM [6] clearly states in article 18 that *“Member States shall ensure that practitioners and the individuals involved in the practical aspects of medical radiological procedures have adequate education, information and theoretical and practical training for the purpose of medical radiological practices, as well as relevant competence in radiation protection”*, there is great heterogeneity in the way each member state applies this in practice, despite the guidance defined by the MEDRAPET [7] (Medical Radiation Protection Education and Training) project. This EU-funded, ESR-coordinated study on the implementation of the Medical Exposure Directive’s requirements within the European Union aimed to improve the implementation of the Medical Exposure Directive provisions related to radiation protection education and training of medical professionals in the EU Member States. As part of the project, a Guidance Document was published in the radiation protection series of the European Commission (No 175) [8].

As concluded in the results of WP7 of the EURAMED rocc-n-roll project [9] “*E&T in RP is of paramount importance for health professionals and researchers to acquire and develop knowledge, skills and competences in the field of RP to protect patients and staff from the dangers arising from the exposure to ionising radiation. Although several projects have been developed in the past years related to E&T in RP, the SWOT analysis showed a clear lack of real and effective implementation of RP principles in daily practice […]. To achieve success, governance structures and strong leadership are key as is the full exploitation of existing resources however equally, appropriate financial support is essential to permit our professions to work collaboratively to achieve a pan European radiation protection training network which is sustainable and accredited across multiple national domains”.*

Although the scope of the EU-REST study is mainly about education, training and workforce availability, an additional attempt was made to characterise the numbers of pieces of medical imaging and radiotherapy equipment in Europe. Despite the efforts made, the level of responses was very limited and, in some cases, contradictory to the data published by EUROSTAT, the OECD and COCIR (European Trade Association representing the medical imaging, radiotherapy, health ICT and electromedical industries). Therefore, any firm conclusions made based on this data are likely be misleading and confounding. Nevertheless, the exercise undertaken is of substantial importance, in particular in calling the attention of the European Commission to the urgent need to develop a strategy to create a centralised repository of medical imaging equipment, with verifiable, reliable and consistent data. This approach would be in line with article 60 of the Directive 2013/51/EURATOM, where it is requested that Member States must ensure *“b) an up-to-date inventory of medical radiological installation is available to competent authority”* [10]. As is the case with the need to establish uniform methods of enumerating workload for the professional groups covered by the EU-REST study, such a repository will need to define exactly how each type of equipment is counted, and how ambiguity will be avoided. While such definitions and methodology are outside the scope of the EU-REST study, it would be a fruitful area for further study and collaborative work in the future, to facilitate ever-closer union among EU Member States in terms of uniform data collection and inter-country comparison.

### Limitations

The EU-REST project included limitations inherent to all projects dependent on surveys, namely a variable (and non-compellable) response rate, and the use of a single language (English) which has the potential of conflicting interpretations of the questions, due to the fact that the great majority of EU countries do not use English as a first language.

It is also important to highlight the fact that the organisations/entities from each Member State which were responsible for replying to these surveys indicated a high level of “survey tiredness”, as there were several EU projects running at the same time, in some cases searching for the same type of information from the same people. We fear that this may have led to incomplete or absent responses from some respondents to some questions. While extensive efforts were made during the cleaning phase of the survey data to fill gaps in supplied responses, these were, inevitably, only of limited success. In the absence of any compellability, no tools were available to the study consortium to supply data which might be desirable, but which were not provided as we followed the pre-defined and pre-agreed methodology of the study.

Furthermore, verification of the accuracy of survey responses was generally not possible, in the absence of objective data against which to check responses (indeed, one of the motivators for this project was the previous absence of such objective data). This particular limitation was ameliorated to some extent by seeking (and in some cases receiving) survey responses from national authorities with specific competence in the areas being interrogated (national authorities etc.).

In Part 3 [5], we will outline the guidelines and recommendations that have emerged from the EU-REST project, with respect to appropriate workforce planning and appropriate education and training standards for radiologists.

The Final Report of the EU-REST project has been published by the European Commission [11] and contains further details of all aspects of the project.

## Supplementary information

ELECTRONIC SUPPLEMENTARY MATERIAL

## Data Availability

European Commission: European Health and Digital Executive Agency, Analysis on workforce availability, education and training needs for the quality and safety of medical applications involving ionising radiation in the EU—Status and recommendations—Final report, Publications Office of the European Union, 2025, 10.2925/2213975.
