# Supplementary material for: Current status of radiologist staffing, education and training in the 27 EU Member States
Source: Insights Imaging. 2025 Mar 15;16:59. doi: 10.1186/s13244-025-01925-7 (PMC11910488; doi:10.1186/s13244-025-01925-7)
Supplement: Supplementary file 1 — ELECTRONIC SUPPLEMENTARY MATERIAL [file 13244_2025_1925_MOESM1_ESM.pdf]

Current status of radiologist staffing, education & training in the 27 EU Member States

ELECTRONIC SUPPLEMENTARY MATERIAL

Insights Imaging (2025) Brady AP, Paulo G, Brkljacic B, Loewe C, Szucsich M, Hierath M.

Supplementary Table S1: Detailed overview of Main Survey responses (prior to data cleaning)

				Answers provided on behalf of						Professions for which the answers were provided							
	Respo n-ses full version	Respon-ses abbr version (Q&S)	**Total respon-ses**	National profes-sional/ scientific soc	National Health Authority	National RP Autho-rity	Licen-sing Autho-rity	Other	Radiolo -gists	Radiation Oncolo-gists	NM Physi-cians	Radio-graphers	RTTs (where indep. from Radio-graphers)	Medical Physi-cists	Other	Combi-ned resp-onse for all Profes-sions	no selec-tion made/ no data provi-ded
**Austria**	7	0	**7**	3	1	0	2	1	3	1	1	1	0	1	0	0	0
**Belgium**	8	1	**9**	5	2	1	0	1	1	2	2	0	2	2	0	0	0
**Bulgaria**	6	1	**7**	6	1	0	0	0	2	0	0	3	1	1	0	0	0
**Croatia**	3	1	**4**	1	0	1	1	1	1	0	1	1	0	0	0	1	0
**Cyprus**	5		**5**	2	1	0	0	2	0	1	0	0	0	4	0	0	0
**Czechia**	6		**6**	5	1	0	0	0	1	1	1	1	0	2	0	0	0
**Denmark**	6		**6**	4	1	0	0	1	1	0	1	1	0	3	0	0	0
**Estonia**	8		**8**	6	1	0	0	1	1	2	1	2	0	1	0	0	1
**Finland**	4	1	**5**	4	0	1	0	0	1	1	0	1	0	2	0	0	0
**France**	5		**5**	5	0	0	0	0	1	0	1	1	0	2	0	0	0
**Germany**	8		**8**	6	0	0	0	2	2	1	1	1	0	3	0	0	0
**Greece**	4		**4**	3	0	1	0	0	1	1	0	1	0	1	0	0	0
**Hungary**	6	2	**8**	5	0	2	0	1	2	1	1	0	1	1	0	2	0
**Ireland**	6		**6**	4	0	0	0	2	1	1	0	1	1	2	0	0	0
**Italy**	15		**15**	10	1	0	0	4	1	7	0	2	3	2	0	0	0
**Latvia**	2		**2**	2	0	0	0	0	0	0	0	1	0	1	0	0	0
**Lithuania**	6	1	**7**	4	0	1	0	2	2	2	0	1	0	2	0	0	0
**Luxembourg**	0	1	**1**	0	0	1	0	0	0	0	0	0	0	0	1	0	0
**Malta**	7		**7**	3	2	0	0	2	1	1	0	3	0	2	0	0	0
**Netherlands**	10	1	**11**	7	0	1	0	3	1	3	1	1	1	3	0	1	0
**Poland**	6		**6**	4	1	1	0	0	2	1	1	1	0	1	0	0	0
**Portugal**	4		**4**	4	0	0	0	0	0	0	1	1	0	2	0	0	0
**Romania**	10		**10**	4	0	1	0	5	2	1	0	0	4	3	0	0	0

**Slovakia**	9	3	**12**	4	0	4	0	4	2	1	1	2	0	5	0	0	1
**Slovenia**	5	2	**7**	5	0	2	0	0	3	1	0	1	0	1	0	0	1
**Spain**	9	1	**10**	6	1	1	0	2	5	2	0	0	0	3	0	0	0
**Sweden**	4	2	**6**	3	1	2	0	0	1	0	1	1	0	3	0	0	0
**Total**	**169**	**17**	**186**	**115**	**14**	**20**	**3**	**34**	**38**	**31**	**15**	**28**	**13**	**53**	**1**	**4**	**3**

The response rates per country per target group addressed (National professional/scientific society, National Health Authority, National RP Authority, Licencing Authority, Other) and per profession are provided in Supplementary Table S2. Multiple responses from one and the same country are counted as 1.

Supplementary Table S2_Response rates for Main Survey (prior to data cleaning)

	Answers provided on behalf of						Professions for which the answers were provided								
	National profes-sional/scien-tific soc.	National Health Authority	National RP Authority	Licensing Authority	Other	% of respon-ding target groups per country	Radiolo-gists	Radiation Oncolo-gists	NM Physicians	Radio-graphers	RTTs (where indep. from Radio-graphers)	Medical Physicists	Other	% of res-ponding professional groups per country	
**Austria**	1	1	0	1	1	80%	1	1	1	1	0	1	0	71%	
**Belgium**	1	1	1	0	1	80%	1	1	1	0	1	1	0	71%	
**Bulgaria**	1	1	0	0	0	40%	1	0	0	1	1	1	0	57%	
**Croatia**	1	0	1	1	1	80%	1	0	1	1	0	0	0	43%	
**Cyprus**	1	1	0	0	1	60%	0	1	0	0	0	1	0	29%	
**Czechia**	1	1	0	0	0	40%	1	1	1	1	0	1	0	71%	
**Denmark**	1	1	0	0	1	60%	1	0	1	1	0	1	0	57%	
**Estonia**	1	1	0	0	1	60%	1	1	1	1	0	1	0	71%	1)
**Finland**	1	0	1	0	0	40%	1	1	0	1	0	1	0	57%	
**France**	1	0	0	0	0	20%	1	0	1	1	0	1	0	57%	
**Germany**	1	0	0	0	1	40%	1	1	1	1	0	1	0	71%	
**Greece**	1	0	1	0	0	40%	1	1	0	1	0	1	0	57%	
**Hungary**	1	0	2	0	1	80%	1	1	1	0	1	1	0	71%	
**Ireland**	1	0	0	0	1	40%	1	1	0	1	1	1	0	71%	

**Italy**	1	1	0	0	1	60%	1	1	0	1	1	1	0	71%	
**Latvia**	1	0	0	0	0	20%	0	0	0	1	0	1	0	29%	
**Lithuania**	1	0	1	0	1	60%	1	1	0	1	0	1	0	57%	
**Luxembourg**	0	0	1	0	0	20%	0	0	0	0	0	0	1	14%	
**Malta**	1	1	0	0	1	60%	1	1	0	1	0	1	0	57%	
**Netherlands**	1	0	1	0	1	60%	1	1	1	1	1	1	0	86%	
**Poland**	1	1	1	0	0	60%	1	1	1	1	0	1	0	71%	
**Portugal**	1	0	0	0	0	20%	0	0	1	1	0	1	0	43%	
**Romania**	1	0	1	0	1	60%	1	1	0	0	1	1	0	57%	
**Slovakia**	1	0	1	0	1	60%	1	1	1	1	0	1	0	71%	1)
**Slovenia**	1	0	1	0	0	40%	1	1	0	1	0	1	0	57%	1)
**Spain**	1	1	1	0	1	80%	1	1	0	0	0	1	0	43%	
**Sweden**	1	1	1	0	0	60%	1	0	1	1	0	1	0	57%	
**Resp. rate per responder type, all countries**	96%	44%	56%	7%	59%		85%	70%	52%	78%	26%	93%	4%		

1) One additional answer was received for "medical doctor" without specification Radiologists/Radiation Oncologists/NM Physicians.

EU-REST

EU-REST Pre-Survey

The EU-REST study aims to provide an analysis of workforce availability, education, and training needs to ensure quality and safety aspects of medical applications involving ionising radiation in the EU. The EU-REST study is part of the SAMIRA Action Plan and is being carried out on behalf of the European Commission. The consortium undertaking this study includes representatives from the European Society of Radiology (ESR), European Society for Radiotherapy and Oncology (ESTRO), European Federation of Organisations For Medical Physics (EFOMP), European Federation of Radiographer Societies (EFRS) and European Association of Nuclear Medicine (EANM).

The study will seek to address the needs for a highly-qualified workforce and proper forecasts of staff requirements by collecting, analysing, and making widely available up-to-date data on staffing, education, and training of the key professional groups in EU Member States. To enable this, the EU-REST study invites you to complete this Pre-Survey to help us identify the relevant authorities/ professional bodies etc. within your country.

Please note: there is no 'save your answers and complete later' function on this survey. Therefore, **we recommend you read the questions offline first** (circulated as a pdf with the link to this survey), gather the information required, and then complete the survey.

Please note: the questions marked with an asterisk (*) are mandatory (i.e. they must be answered before you can finish the survey). If you do not know the answer, please state this in the text box provided.

Please note: in order to comply with GDPR rules, please ensure you have the permission of the relevant contact persons to share their details. Alternatively, please provide links to where the relevant details may be found online.

Please note: your contact details are requested. Your details will not be shared with any other parties and will only be used to contact you in relation to the EU-REST project for the purpose of following up on any questions the EU-REST study team might have regarding your answers.

Part 1: Demographics:

Title

[ ]

[ ]

* What is your name?

First name [ ]

Last name [ ]

* Email address

[ ]

* On behalf of what country are you responding to this Pre-Survey?

The next question asks what profession you are responding on behalf of. As names and categorisation of professions can vary between countries, please note the additional points below:

Radiographers are known by a variety of terms, including radiology technologists.

Radiation Therapist are also known as RTT, radiotherapy technologist or therapeutic radiographer in some countries, distinct from Radiation Oncologists.

Medical Physicists, may include Radiation Protection Advisors & Medical Physics Experts, depending on categorisation in your country.

* On behalf of what profession are you responding?

- Medical doctor (Radiologist, Radiation Oncologist, and/or Nuclear Medicine physician)
- Radiographer/Radiation Therapist
- Medical Physicist
- Other profession using ionising radiation (focusing on high-dose procedures), please specify:

EU-REST

EU-REST Pre-Survey

Part 2: Pre-survey

* Which profession are the answers below for?

- [ ] Radiologist
- [ ] Radiation Oncologist (also known as clinical oncologists and, in some countries, Radiotherapists)
- [ ] Nuclear Medicine physician

* Please identify and provide contact details for the relevant authorities/professional bodies etc. within your country with competence in and responsibility for **setting the curriculum for medically-qualified specialty trainees (residents)** i n the profession indicated.
If a different body determines the numbers of places available for such trainees, please give details of that body by selecting 'Add contact' and explaining this under 'other comments'.

Name of authority/professional body in charge ______

Website ______

Contact person first name ______

Contact person last name ______

Contact's email address ______

Contact person's job title/role within authority/body ______

Other comments ______

In case you are responding for more than one profession, please indicate which profession your answers to this question are for ______

* Click here if you wish to add another contact you consider relevant

- ( ) Add contact
- ( ) Move on to next question

EU-REST

EU-REST Pre-Survey

Please identify and provide contact details for the relevant authorities/professional bodies etc. within your country with competence in and responsibility for the following:

* **Setting standards (i.e. duration of training, competencies to be achieved, exams to be passed etc.) for education and training** (if this body is different from the body outlined in the last question.

Name of authority/professional body in charge

Website

Contact person first name

Contact person last name

Contact's email address

Contact person's job title/role within authority/body

Other comments

In case you are responding for more than one profession, please indicate which profession your answers to this question are for

* Click here if you wish to add another contact you consider relevant

- Add contact
- Move on to next question

EU-REST Pre-Survey

Please identify and provide contact details for the relevant authorities/professional bodies etc. within your country with competence in and responsibility for the following:

*** Determining the number of vacancies for medically-qualified specialty trainees (residents per year).**

Name of authority/professional body in charge	
Website	
Contact person first name	
Contact person last name	
Contact's email address	
Contact person's job title/role within authority/body	
Other comments	
In case you are responding for more than one profession, please indicate which profession your answers to this question are for	

* Click here if you wish to add another contact you consider relevant

- Add contact
- Move on to next question

EU-REST

EU-REST Pre-Survey

Please identify and provide contact details for the relevant authorities/professional bodies etc. within your country with competence in and responsibility for the following:

* **Initial licensing/certification of those permitted to practice independently as a specialist.**
If a different body is responsible for managing subsequent maintenance of licensing/certification (recertification), please give details of that body by selecting 'Add contact' and explaining this under 'other comments'.

Name of authority/professional body in charge

Website

Contact person first name

Contact person last name

Contact's email address

Contact person's job title/role within authority/body

Other comments

In case you are responding for more than one profession, please indicate which profession your answers to this question are for

* Click here if you wish to add another contact you consider relevant

- Add contact
- Move on to next question

EU-REST

EU-REST Pre-Survey

Please identify and provide contact details for the relevant authorities/professional bodies etc. within your country with competence in and responsibility for the following:

*** Determining the necessary continuing professional development / continuing education for maintenance of licensing as a specialist.**

If a different body is responsible for determining the necessary continuing education for radiation protection, please give details of that body by selecting 'Add contact' and explaining this under 'other comments'.

Name of authority/professional body in charge

Website

Contact person first name

Contact person last name

Contact's email address

Contact person's job title/role within authority/body

Other comments

In case you are responding for more than one profession, please indicate which profession your answers to this question are for

* Click here if you wish to add another contact you consider relevant

- Add contact
- Move on to next question

EU-REST

EU-REST Pre-Survey

Please identify and provide contact details for the relevant authorities/professional bodies etc. within your country with competence in and responsibility for the following:

* **Collating workload and activity data** (i.e. measurements used to determine amount of work done, e.g. numbers of patients treated, numbers of imaging studies performed and/or reported etc.

Name of authority/professional body in charge

Website

Contact person first name

Contact person last name

Contact's email address

Contact person's job title/role within authority/body

Other comments

In case you are responding for more than one profession, please indicate which profession your answers to this question are for

* Click here if you wish to add another contact you consider relevant

- Add contact
- Move on to next question

EU-REST

EU-REST Pre-Survey

Please identify and provide contact details for the relevant authorities/professional bodies etc. within your country with competence in and responsibility for the following:

*** Determining health service staffing levels**

Name of authority/professional body in charge

Website

Contact person first name

Contact person last name

Contact's email address

Contact person's job title/role within authority/body

Other comments

In case you are responding for more than one profession, please indicate which profession your answers to this question are for

* Click here if you wish to add another contact you consider relevant

- Add contact
- Move on to next question

EU-REST Pre-Survey

Some other medical specialists and professions utilise ionising radiation in the performance of their work. Some of these confer relatively low radiation doses on patients (e.g. dentists). Examples of usage with the potential for high radiation dose include interventional cardiology, gastroenterology, endovascular intervention, and some surgical specialties (e.g. urology, orthopaedic and trauma surgery, neurosurgery). Please identify and provide contact details for the relevant authorities/professional bodies etc. within your country with competence in and responsibility for the following:

Determining the training necessary and licensing (if applicable) for the use of ionising radiation by medical specialists in other professions using ionising radiation (focusing on high-dose procedures).

Name of authority/professional body in charge

Website

Contact person first name

Contact person last name

Contact's email address

Contact person's job title/role within authority/body

Other comments

An element of this project will involve consultation with stakeholders about the draft recommendations on education, training and workforce which will be written. Please give contact details for relevant stakeholders you believe should be consulted within your country.

Name of authority/professional body in charge

Website

Contact person first name

Contact person last name

Contact's email address

Contact person's job title/role within authority/body

Other comments

* Do you need to add another stakeholder?

- ◯ Yes
- ◯ No

The EU-REST study aims to provide an analysis of workforce availability, education, and training needs to ensure quality and safety aspects of medical applications involving ionising radiation in the EU. The EU-REST study is part of the SAMIRA Action Plan (europa.eu) and is being carried out on behalf of the European Commission. The consortium undertaking this study includes representatives from the European Society of Radiology (ESR), European Society for Radiotherapy and Oncology (ESTRO), European Federation of Organisations For Medical Physics (EFOMP), European Federation of Radiographer Societies (EFRS) and European Association of Nuclear Medicine (EANM).
The study will seek to address the needs for a highly-qualified workforce and proper forecasts of staff requirements by collecting, analysing, and making widely available up-to-date data on staffing, education, and training of the key professional groups in EU Member States. To enable this, the EU-REST study invites you to complete this Survey and provide information for the profession(s) you indicate in your selected country.

Once you have gathered the relevant information (see notes below), this Survey should take approximately 20-30 minutes to complete.

Please note: we recommend you read the questions offline first (the questions were circulated as a pdf with the link to this survey), gather the information required, and then complete the survey. The offline pdf document is intended as a representative version of the survey – some questions in the online version may change slightly or be skipped depending on answers given e.g. according to profession indicated.

Please note: the questions marked with an asterisk (*) are mandatory (i.e. they must be answered before you can finish the survey).

Section 0: Demographics

Please complete the following. Your details will not be shared with any other parties and will only be used to contact you in relation to the EU-REST project for the purpose of following up on any questions the EU-REST study team might have regarding your answers.

1. Title

* 2. What is your name?

First name

Last name

* 3. Email address

* 4. On behalf of what country are you responding to this Survey?

* 5. On behalf of what organisation / body are your answers provided?

- ◯ National Professional / Scientific Society
- ◯ National Health Authority
- ◯ National Radiation Protection Authority
- ◯ Licensing Authority (e.g. Medical Council or Chamber etc.)
- ◯ Other (please specify)

* 6. What is the name of the body / organisation for which your answers are provided?

Section 0: Demographics

If you wish to provide answers for more than one profession, please select the first profession for which you will answer. Once you have completed the survey for that profession, you will be given the option to answer for (an) additional profession(s). If your answers will be the same for multiple professions, we recommend that you save your free-text answers in a separate document so that you may copy and paste them.

* 7. For what profession are you giving answers?

- ◯ **A. Medical Doctors**
  i) Radiologists
  ii) Radiation Oncologists (*also known as Clinical Oncologists and, in some countries, Radiotherapists, distinct from (B.iii) and (C) below*)
  iii) Nuclear Medicine physicians

- ◯ **B. Radiographers** (*known by a variety of terms, including Technologists etc.*)
  i) Diagnostic & Interventional Radiology (*including Ultrasound, where this is performed by Radiographers*)
  ii) Nuclear Medicine
  iii) Radiation Therapists / Radiotherapy / Radiation Oncology *(if this group of workers fall under the category of Radiographers in your country)*

- ◯ **C. Radiation Therapists** (*known as RTT, Radiotherapy Technologist, RTT Nurse or Therapeutic Radiographer in some countries, distinct from (A.ii) above*) *(if this group of workers are independent from the category of Radiographers - as listed in (B.iii) above - in your country)*
  i) Radiotherapy / Radiation Oncology

- ◯ **D. Medical Physicists** (*including Radiation Protection Advisors, Radiation Protection Experts & Medical Physics Experts, depending on categorisation in each country*)
  i) Diagnostic & Interventional Radiology
  ii) Nuclear Medicine
  iii) Radiotherapy / Radiation Oncology

- ◯ **E. Other professions using ionising radiation** (*focusing on high-dose procedures*): Some other medical specialists and professions utilise ionising radiation in the performance of their work.

Section A1: Education & Training (Medical Doctors)

Primary professional education/training (before certification for independent practice)

* 8. Please select the specialty for which you will provide answers. If you wish to provide answers for more than one specialty, please select the *first* profession for which you will provide answers.

- ○ Radiologists
- ○ Radiation Oncologists
- ○ Nuclear Medicine Physicians

* 9. Before entering training for the profession, must a candidate meet specific criteria (e.g. pass certain exams or complete certain qualifications)?

- ○ Yes
- ○ No
- ○ Don't know

Section A1: Education & Training (Medical Doctors)

Primary professional education/training (before certification for independent practice)

10. What are the minimum entry criteria for entry into training for the profession?

Section A1: Education & Training (Medical Doctors)

Primary professional education/training (before certification for independent practice)

11. Is a period of prior training / practice in a different clinical specialty (or general clinical skills), after graduation from medical school, required before commencement of specialty training in the selected medical discipline?

- ◯ Yes
- ◯ No

Section A1: Education & Training (Medical Doctors)

Primary professional education/training (before certification for independent practice)

12. How long is that period?

Section A1: Education & Training (Medical Doctors)

Primary professional education/training (before certification for independent practice)

13. What professional / educational / regulatory body(ies) determine(s) the educational curriculum for Medical Doctors?

14. At what level(s) is the curriculum used determined (select all that apply)?

- [ ] Locally
- [ ] Nationally
- [ ] Internationally

* 15. Which of the following topics does the training programme / curriculum include (select all that apply)?

- [ ] Radiation protection for staff
- [ ] Radiation protection for patients / general public
- [ ] Radiation protection legislation
- [ ] Quality & safety management
- [ ] Good research practice
- [ ] Pallative care (*if answering for Radiation Oncology*)
- [ ] None of these options
- [ ] Don't know
- [ ] Artificial intelligence theory and applications (please give details of scope and content of relevant part of curriculum below)

Section A1: Education & Training (Medical Doctors)

Primary professional education/training (before certification for independent practice)

* 16. Is the radiation protection curriculum defined by a different professional / educational / regulatory body?

- Yes
- No
- Don't know

Section A1: Education & Training (Medical Doctors)

Primary professional education/training (before certification for independent practice)

17. Which professional / educational / regulatory body defines the radiation protection curriculum?

Section A1: Education & Training (Medical Doctors)

Primary professional education/training (before certification for independent practice)

18. What professional / educational body(ies) provide(s) education for the selected medical specialty (i.e. is education provided solely at local (e.g. university) level, or is there a larger over-seeing body)?

19. How is this training delivered and assessed (select all that apply)?

- [ ] Locally
- [ ] Nationally
- [ ] Internationally

20. How are training centres selected?

By what body is selection performed?

* 21. Are criteria established to select training centres?

- ( ) Yes
- ( ) No
- ( ) Don't know

Section A1: Education & Training (Medical Doctors)

Primary professional education/training (before certification for independent practice)

22. Please provide details of (e.g. links to) the criteria

Section A1: Education & Training (Medical Doctors)

Primary professional education/training (before certification for independent practice)

23. Do selected training centres have the freedom to vary the training curriculum, or must a uniform curriculum be followed?

- ◯ Selected centres are free to vary the curriculum
- ◯ Selected centres can vary part (but not all) of the curriculum
- ◯ A fixed curriculum must be followed
- ◯ Don't know

* 24. Are training centres formally certified / assessed / audited?

- ◯ Yes
- ◯ No
- ◯ Don't know

Section A1: Education & Training (Medical Doctors)

Primary professional education/training (before certification for independent practice)

25. How frequently are training centres formally assessed?

Section A1: Education & Training (Medical Doctors)

Primary professional education/training (before certification for independent practice)

26. What is the minimum duration of training in the specialty (excluding clinical training prior to specialty training)? Please provide answers **in years** as a decimal (e.g. 2.0, 0.25, 1.5 etc.)

27. Please indicate the estimated average percent of time during specialty training spent on theoretical (classroom teaching etc.) and practical (patient contact, practical work & supervised service delivery):

100% theoretical | 50/50 | 100% practical

28. Is a uniform method of completion of training applied in your country?

- Yes
- No

* 29. How is completion of specialty (full professional) training achieved (select all that apply)?

- Examination
- Defined time period
- Achievement of defined competencies
- Don't know
- Other (please specify)

Section A1: Education & Training (Medical Doctors)

Primary professional education/training (before certification for independent practice)

30. If completion of specialty (full professional) training is achieved through examination, who sets and administers the examination?

Section A1: Education & Training (Medical Doctors)

Primary professional education/training (before certification for independent practice)

31. Within training for the profession / specialty, what are the specific training elements relating to radiation protection which must be fulfilled (select all that apply)?

- [ ] Atomic structure, X-ray production and interaction of radiation
- [ ] Nuclear structure and radioactivity
- [ ] Radiological quantities and units
- [ ] Physical characteristics of X-ray systems
- [ ] Fundamentals of radiation detection
- [ ] Fundamentals of radiobiology, biological effects of radiation
- [ ] Risks of cancer and hereditary disease and effective dose
- [ ] Risks of deterministic / tissue effects
- [ ] General principles of radiation protection
- [ ] Operational radiation protection
- [ ] Particular patient radiation protection aspects
- [ ] Particular staff radiation protection aspects
- [ ] Typical doses from diagnostic procedures
- [ ] Risks from foetal exposure to ionising radiation
- [ ] Quality control and quality assurance in radiation protection
- [ ] National regulations and international standards
- [ ] Dose management of pregnant patients
- [ ] Dose management of pregnant staff
- [ ] The process of justification of imaging examinations
- [ ] Management of accidents/unintentional exposures
- [ ] Don't know
- [ ] Other (please specify)

32. Within training for the profession / specialty, how much training time is dedicated to radiation protection?

* 33. Is specific certification required in radiation protection (separate from full certification to practice in the professional group)?

- ◯ Yes
- ◯ No
- ◯ Don't know

Section A1: Education & Training (Medical Doctors)

Primary professional education/training (before certification for independent practice)

34. Who provides this certification?

- ○ Health Authority
- ○ Radiation Protection Authority
- ○ Professional Society
- ○ Don’t know
- ○ Other (please specify)

35. Is such certification required for independent practice?

- ○ Yes
- ○ No

Section A1: Education & Training (Medical Doctors)

Primary professional education/training (before certification for independent practice)

36. What, if any, higher qualifications (beyond those given previously) are available to practitioners within the specialty (select all that apply)?

- [ ] None
- [ ] Specialised courses
- [ ] Postgraduate courses
- [ ] Master courses
- [ ] PhD courses
- [ ] Post doc courses
- [ ] Don't know
- [ ] Other (please specify)

* 37. Are any qualifications required to complete training to become a full professional in the specialty, or higher qualifications available to practitioners within the profession, mapped against international qualification standards?

- ( ) Yes
- ( ) No
- ( ) Don't know
- ( ) Not applicable

Section A1: Education & Training (Medical Doctors)

Primary professional education/training (before certification for independent practice)

38. At what level (select all that apply)?

- [ ] Licentiate or Bachelors
- [ ] Masters
- [ ] Doctorate
- [ ] Don't know
- [ ] Other (please specify)

Section A1: Education & Training (Medical Doctors)

Primary professional education/training (before certification for independent practice)

* 39. Is additional training beyond the minimum number of training years specified in your earlier answer undertaken before independent practice?

- Yes
- No
- Sometimes, but it is not mandatory
- Don't know

Section A1: Education & Training (Medical Doctors)

Primary professional education/training (before certification for independent practice)

40. Please give details (e.g. after completion of the minimum period of specialty training for independent practice, is further training commonly undertaken, such as medical specialty fellowship training?)

41. Is such additional training a requirement (or, if not a requirement, an expectation) before a person would be appointed to a post allowing independent practice?

- Yes
- No
- Don't know

42. Approximately what percentage of trainees undertake this additional training?

Section A1: Education & Training (Medical Doctors)

Primary professional education/training (before certification for independent practice)

43. How are trainee numbers determined / limited (select all that apply)?

- [ ] Central government
- [ ] Local university or hospital
- [ ] Don't know
- [ ] Other (please specify)

44. How are trainees funded / paid (select all that apply)?

- [ ] Central government
- [ ] Local university or hospital
- [ ] Trainees pay for their training
- [ ] Trainees not paid
- [ ] Don't know
- [ ] Other (please specify)

45. How are trainees selected (select all that apply)?

- [ ] Competitive exam
- [ ] Personal interview
- [ ] Selection based on CV
- [ ] Motivation letter
- [ ] Don't know
- [ ] Other (please specify)

Section A2: Education & Training (Medical Doctors)

Continuing professional development/Continuing education (CPD/CE)

* 46. Following completion of primary education & training and entry into independent practice, is continuing professional development / continuing education mandatory for the selected medical specialty?

- ◯ Yes
- ◯ No
- ◯ Don't know

Section A2: Education & Training (Medical Doctors)

Continuing professional development/Continuing education (CPD/CE)

* 47. Is voluntary CPD/CE encouraged?

- ◯ Yes
- ◯ No
- ◯ Don't know

Section A2: Education & Training (Medical Doctors)

Continuing professional development/Continuing education (CPD/CE)

48. If continuing professional development / continuing education is mandatory, what form does this take?

(A) A single-year requirement (credits must be accumulated within a single year); or, (B) A multi-year cycle (credits must be accumulated over a number of years)?	
(A) Self-directed; or, (B) Provided by outside bodies/agencies? (C) Both	

Other (please specify)

* 49. Is participation in continuing professional development / continuing education audited?

- Yes
- No
- Don't know

Section A2: Education & Training (Medical Doctors)

Continuing professional development/Continuing education (CPD/CE)

50. How is it audited

Internally within the employing organisation?	
By an external regulator?	

51. If it is audited by an external regulator, what regulator?

Section A2: Education & Training (Medical Doctors)

Continuing professional development/Continuing education (CPD/CE)

* 52. Is re-certification (i.e. formal re-certification of qualifications permitting practice/work) to maintain professional qualifications undertaken?

- ○ Yes
- ○ No
- ○ Don't know

Section A2: Education & Training (Medical Doctors)

Continuing professional development/Continuing education (CPD/CE)

53. At what interval is re-certification undertaken?

54. How is re-certification undertaken?

* 55. Is specific continuing education **in radiation protection** mandatory?

- Yes
- No
- Don't know

Section B: Workforce availability (Medical Doctors)

* 238. Is a central register for the selected medical specialty maintained by a central body in your country?

- Yes
- No
- Don't know

Section B: Workforce availability (Medical Doctors)

239. What is the name of that body?

Section B: Workforce availability (Medical Doctors)

240. How many practitioners of the selected specialty are licensed to practise in your country?

241. How many practitioners of the selected specialty are actually in practice (excluding retired, inactive, abroad etc.) in your country?

* 242. Is the proportion of staff in (professional group) working full-time or less than full-time in your country known?

- Yes
- No
- Don't know

Section B: Workforce availability (Medical Doctors)

243. Please give details, number of full-time equivalents etc.

Section B: Workforce availability (Medical Doctors)

244. How many full-time equivalents are in practice?

Section B: Workforce availability (Medical Doctors)

245. Is it possible for a professional to work in both public and private centres?

- Yes
- No
- Don't know

* 246. Is information available about the numbers in practice in public healthcare and private practice?

- Yes
- No
- Don't know

Section B: Workforce availability (Medical Doctors)

247. Please provide the number for practice in public healthcare.

248. Please provide the number for practice in private healthcare.

Section B: Workforce availability (Medical Doctors)

249. What % of professionals work in both public AND private centres?

Section B: Workforce availability (Medical Doctors)

250. What is the gender-profile mix?

0% Male — 100% Male

251. What is the age-profile mix? Please enter only a number to indicate the percentage of professionals in each age group. Please ensure the total equals 100.

<30

31-40

41-50

51-60

>61

252. Please supply, if available, data about the proportion of sanctioned posts which are unfilled.

253. Please supply, if available, data about the numbers of planned retirements in the next 5, 10 and 20 years.

* 254. Is there reliance on graduates from the selected specialty from overseas (non-national and/or from outside the EU-27)) for workforce maintenance?

- Yes
- No
- Don't know

Section B: Workforce availability (Medical Doctors)

255. As a percentage of the total workforce, to what extent is the workforce maintained by reliance on graduates from overseas?

Section B: Workforce availability (Medical Doctors)

256. What proportion of new graduates in the selected specialty are absorbed by your country’s staffing needs?

Section B: Workforce availability (Medical Doctors)

* 257. If staff shortages for the selected specialty exist in your country, are staff who have not completed full formal training employed for independent (unsupervised) service delivery?

- ◯ Yes
- ◯ No
- ◯ Don't know

Section B: Workforce availability (Medical Doctors)

258. What proportion of active staff in practice **do not** fulfil full training requirements?

Section B: Workforce availability (Medical Doctors)

* 259. Is such reliance on unqualified or under-qualified staff a short-term or permanent arrangement?

- ◯ Short-term
- ◯ Permanent
- ◯ Not applicable
- ◯ Don't know

Section C: Workforce planning (Medical Doctors)

348. What is the mechanism to increase numbers of the selected specialty in an institution (i.e. a specific centre. E.g. How does a hospital increase its numbers of doctors within the selected specialty etc.)? Please give details of the following:

What is the source of the funding needed for additional practitioners?

How long does it typically take to recruit a new practitioner (from decision to recruit to starting work)?

Are sufficient qualified practitioners available within your country to fill all available vacancies?

* 349. **If you are providing answers for Radiology:** is data available for national equipment availability relevant for **Diagnostic Radiology**?

- Yes
- No
- Don't know
- Not applicable

Section C: Workforce planning (Medical Doctors)

350. Please provide the numbers of equipment for the following categories in your country:

CT scanners	
MRI scanners	
US machines	
Diagnostic radiography units	
Mammography units	
Mobile radiology units	
Angiographic / interventional suites	

351. Please indicate the **year** from which the numbers are recorded.

CT scanners	
MRI scanners	
US machines	
Diagnostic radiography units	
Mammography units	
Mobile radiology units	
Angiographic / interventional suites	

Section C: Workforce planning (Medical Doctors)

* 352. **If you are providing answer for Radiology or Nuclear Medicine:** is data available for national equipment availability relevant for **Nuclear Medicine**?

- ◯ Yes
- ◯ No
- ◯ Don't know
- ◯ Not applicable

Section C: Workforce planning (Medical Doctors)

353. Please provide the numbers of equipment for the following categories in your country:

Gamma cameras	
SPECT cameras	
SPECT/CT cameras	
PET scanners	
PET/CT scanners	
PET/MR	
Cyclotrons	

354. Please indicate the **year** from which the numbers are recorded.

Gamma cameras	
SPECT cameras	
SPECT/CT cameras	
PET scanners	
PET/CT scanners	
PET/MR	
Cyclotrons	

Section C: Workforce planning (Medical Doctors)

* 355. **If you are providing answers for Radiation Oncology:** is data available for national equipment availability relevant for **Radiation Oncology**?

- ◯ Yes
- ◯ No
- ◯ Don't know
- ◯ Not applicable

Section C: Workforce planning (Medical Doctors)

356. Please provide the numbers of equipment for the following categories in your country:

Linear accelerators	
Brachytherapy / intraoperative units	
Particle therapy units	
Cobalt units	
kV therapy / orthovoltage units	

357. Please indicate the **year** from which the numbers are recorded.

Linear accelerators	
Brachytherapy / intraoperative units	
Particle therapy units	
Cobalt units	
kV therapy / orthovoltage	

Section C: Workforce planning (Medical Doctors)

358. Is the data provided for pieces of equipment only for public healthcare services, or are private facilities also included?

- Public healthcare services only
- Public and private healthcare services
- Not applicable

Section C: Workforce planning (Medical Doctors)

* 359. Is data available for national workload for elements relevant for the professional group for which you are answering?

- Yes
- No
- Don't know

Section C: Workforce planning (Medical Doctors)

Please provide numerical data for the national workload relevant to the professional group for which you are responding.

360. **Radiology**

No. of Plain radiographs	
No. of Ultrasound studies	
No. of CT studies	
No. of MR studies	
No. of Interventional procedures	

361. **Radiation Oncology**

No. of Radiotherapy treatments	
No. of Patients imaged / treated	
No. of Treatment fractions	

362. **Nuclear Medicine**

No. of Diagnostic Nuclear Medicine studies	
No. of Nuclear Medicine treatments	

Section C: Workforce planning (Medical Doctors)

* 363. Is data available for the trend over time for the numbers given for national equipment availability?

- ◯ Yes
- ◯ No
- ◯ Don't know

Section C: Workforce planning (Medical Doctors)

364. Please give details

Section C: Workforce planning (Medical Doctors)

* 365. Is there any benchmarking method in use to match workforce numbers to workload / activity / equipment availability?

- ◯ Yes
- ◯ No
- ◯ Don't know

Section C: Workforce planning (Medical Doctors)

366. Please give references / sources for the benchmarking methods.

* 367. Does the benchmarking take into account activities such as management, teaching, research, patient consultation etc.?

- ◯ Yes
- ◯ No
- ◯ Don't know

Section C: Workforce planning (Medical Doctors)

368. Please provide details

Section C: Workforce planning (Medical Doctors)

* 369. Is there any defined standard used to determine workforce numbers in the selected specicalty in your country?

- ◯ Yes
- ◯ No
- ◯ Don't know

Section C: Workforce planning (Medical Doctors)

370. Please give reference(s) / source(s) for the standards.

Section D: Quality and Safety (Medical Doctors)

427. Is there a mechanism to assess Quality & Safety in the provision of healthcare involving ionising radiation in your country?

- ◯ Yes
- ◯ No
- ◯ Don't know

Section D: Quality and Safety (Medical Doctors)

428. Please give details about the organisation in charge of assessing Quality and Safety in your country.

Section D: Quality and Safety (Medical Doctors)

* 429. What specific measures are required by legislation (select any that apply)?

- [ ] Regulatory audit
- [ ] Internal Clinical audit
- [ ] External Clinical Audit
- [ ] Formal quality management system reporting to a regulatory/inspection agency
- [ ] Facility inspection by regulatory agencies
- [ ] A national system to report errors
- [ ] None/Not applicable
- [ ] Don't know
- [ ] Other (please specify)

430. What categories of staff in your country are responsible for notifying the competent authority of the occurrence of any significant event resulting or liable to result in the exposure of an individual to ionising radiation beyond the operational limits or conditions of operation specified in authorising requirements (CD 2013/59/Euratom article 96 (b)) (select all that apply)?

- [ ] Radiation protection officer
- [ ] Radiation protection expert
- [ ] MPE
- [ ] RTT
- [ ] Radiographers
- [ ] Medical Doctor Specialist
- [ ] Patient
- [ ] Nurses
- [ ] Quality Manager
- [ ] Don't know
- [ ] Other (please specify)

431. What categories of staff in your country are responsible for reporting to an Incident Learning system (ILS) events involving, or potentially involving, accidental or unintended medical exposures (CD 2013/59/Euratom article 96 (a)) (select all that apply)?

- [ ] Radiation protection officer
- [ ] Radiation protection expert
- [ ] MPE
- [ ] RTT
- [ ] Radiographers
- [ ] Medical Doctor Specialist
- [ ] Patient
- [ ] Nurses
- [ ] Quality Manager
- [ ] Don't know
- [ ] Other (please specify)

432. What categories of staff in your country are responsible for the analysis of events involving, or potentially involving, accidental or unintended medical exposures (CD 2013/59/Euratom article 96 (a)) (select all that apply)?

- [ ] Radiation protection officer
- [ ] Radiation protection expert
- [ ] MPE
- [ ] RTT
- [ ] Radiographers
- [ ] Medical Doctor Specialist
- [ ] Patient
- [ ] Nurses
- [ ] Quality Manager
- [ ] Don't know
- [ ] Other (please specify)

Section E (Medical Doctors)

457. Please include references / links to any documents from your country offering guidance and / or internal standards for the items being surveyed in Sections A-D above.

* 458. Would you like to provide answers for another profession?

- ◯ Yes
- ◯ No
